# The Rat Genome Database 2015: genomic, phenotypic and environmental variations and disease

**DOI:** 10.1093/nar/gku1026

**Published:** 2014-10-29

**Authors:** Mary Shimoyama, Jeff De Pons, G. Thomas Hayman, Stanley J.F. Laulederkind, Weisong Liu, Rajni Nigam, Victoria Petri, Jennifer R. Smith, Marek Tutaj, Shur-Jen Wang, Elizabeth Worthey, Melinda Dwinell, Howard Jacob

**Affiliations:** 1 Human and Molecular Genetics Center, Medical College of Wisconsin, Milwaukee, WI 53226, USA; 2 Department of Surgery, Medical College of Wisconsin, Milwaukee, WI 53226, USA; 3 Department of Pediatrics, Medical College of Wisconsin, Milwaukee, WI 53226, USA; 4 Department of Physiology, Medical College of Wisconsin, Milwaukee, WI 53226, USA

## Abstract

The Rat Genome Database (RGD, http://rgd.mcw.edu) provides the most comprehensive data repository and informatics platform related to the laboratory rat, one of the most important model organisms for disease studies. RGD maintains and updates datasets for genomic elements such as genes, transcripts and increasingly in recent years, sequence variations, as well as map positions for multiple assemblies and sequence information. Functional annotations for genomic elements are curated from published literature, submitted by researchers and integrated from other public resources. Complementing the genomic data catalogs are those associated with phenotypes and disease, including strains, QTL and experimental phenotype measurements across hundreds of strains. Data are submitted by researchers, acquired through bulk data pipelines or curated from published literature. Innovative software tools provide users with an integrated platform to query, mine, display and analyze valuable genomic and phenomic datasets for discovery and enhancement of their own research. This update highlights recent developments that reflect an increasing focus on: (i) genomic variation, (ii) phenotypes and diseases, (iii) data related to the environment and experimental conditions and (iv) datasets and software tools that allow the user to explore and analyze the interactions among these and their impact on disease.

## INTRODUCTION

The Rat Genome Database (RGD) has been the premier resource for rat genetic, genomic and phenotypic data since its creation in 1999. The rat is an important animal model for pharmacology, toxicology, physiology and pathology (1). Because of the rat's utility in diverse studies and its use in studying human disease, RGD has continued to attract increasing numbers of clinical and model organism researchers; in the past year, RGD recorded nearly 125 000 users from 188 countries and territories. While RGD remains dedicated to validating, cataloging and assigning official nomenclature to rat genomic elements, it also incorporates mouse and human genes, quantitative trait loci (QTL) and simple sequence length polymorphisms (SSLPs) both in reports and genome tools for its diverse community of users (Table 1). Individual rat, mouse and human Genome Browsers are maintained and updated with new assemblies in each organism and syntenic tracks on the browsers allow users to move between organisms with a single click.

**Table 1.** The total number of records for genes, transcripts, QTL and SSLPs across the three species available at RGD

	Rat	Mouse	Human
Genes	53 345	47 975	36 393
Protein coding genes	29 682	30 493	19 599
Pseudogenes	14 061	10 531	9366
Transcripts	108 875	93 031	98 691
QTL	2163	4045	1911
SSLPs	50 467	80 692	321 013

Since in some cases gene records are based on assembly-specific gene predictions, the total number of gene records is higher than the per-assembly count of genes.

Much of the value that RGD offers its users can be found in the functional information annotated to genomic elements. Functional annotations are acquired through both manual curation of literature and automated data pipelines that import manual annotations from other sources (2). This combined approach allows RGD to provide a comprehensive functional profile of the genome (Table 2). The large number of rat strains created through inbreeding and genome manipulation provide researchers with a wide variety of models for their specific area of research (Table 3) and RGD maintains comprehensive strain reports including information on origin, source and associated QTL. This resource has increased in value as strain-specific phenotypic and genomic profiles have been generated in the past several years as described below. RGD's multiple innovative software tools and resources, including multiple Genome Browsers (3,4), Disease Portals (4,5), Ontology Browser (6), PhenoMiner (7,8) and Pathway Portal (9,10) make it an extensive research platform and provide navigation among multiple data types and opportunities for data analysis.

**Table 2.** The numbers and types of functional annotations to rat, mouse and human genes at RGD, and the total number of annotations to all objects for each species and ontology as of September 2014

	Rat genes annotated	Total rat annotations	Human genes annotated	Total human annotations	Mouse genes annotated	Total mouse annotations
GO molecular function	17 220	108 784	15 963	95 693	22 515	106 363
GO biological process	16 963	172 419	16 348	138 415	22 950	133 500
GO cellular component	17 224	95 885	17 554	101 969	22 927	91 936
Pathway ontology	5362	18 047	5198	16 699	5267	16 567
CHEBI	20 451	783 074	19 990	821 065	20 432	822 842
RGD disease	3951	47 219	4496	90 364	3868	42 645
Mammalian/human phenotype	1413	6429	3208	52 576	7494	211 170

**Table 3.** The numbers and types of rat strains cataloged at RGD as of September 2014

Total	2998
Inbred	706
Consomic	91
Congenic	1146
Mutant	643
Transgenic	188
Coisogenic	13
Recombinant inbred	131
Segregating inbred	13
Advanced intercross	4
Outbred	53
Hybrid	4
Conplastic	1
Wild	5

With the development of new genomic modification technologies for the rat, the numbers of congenic, mutant and transgenic strains have grown rapidly.

## GENOMIC VARIATION

### Variation data

**Figure 1.**
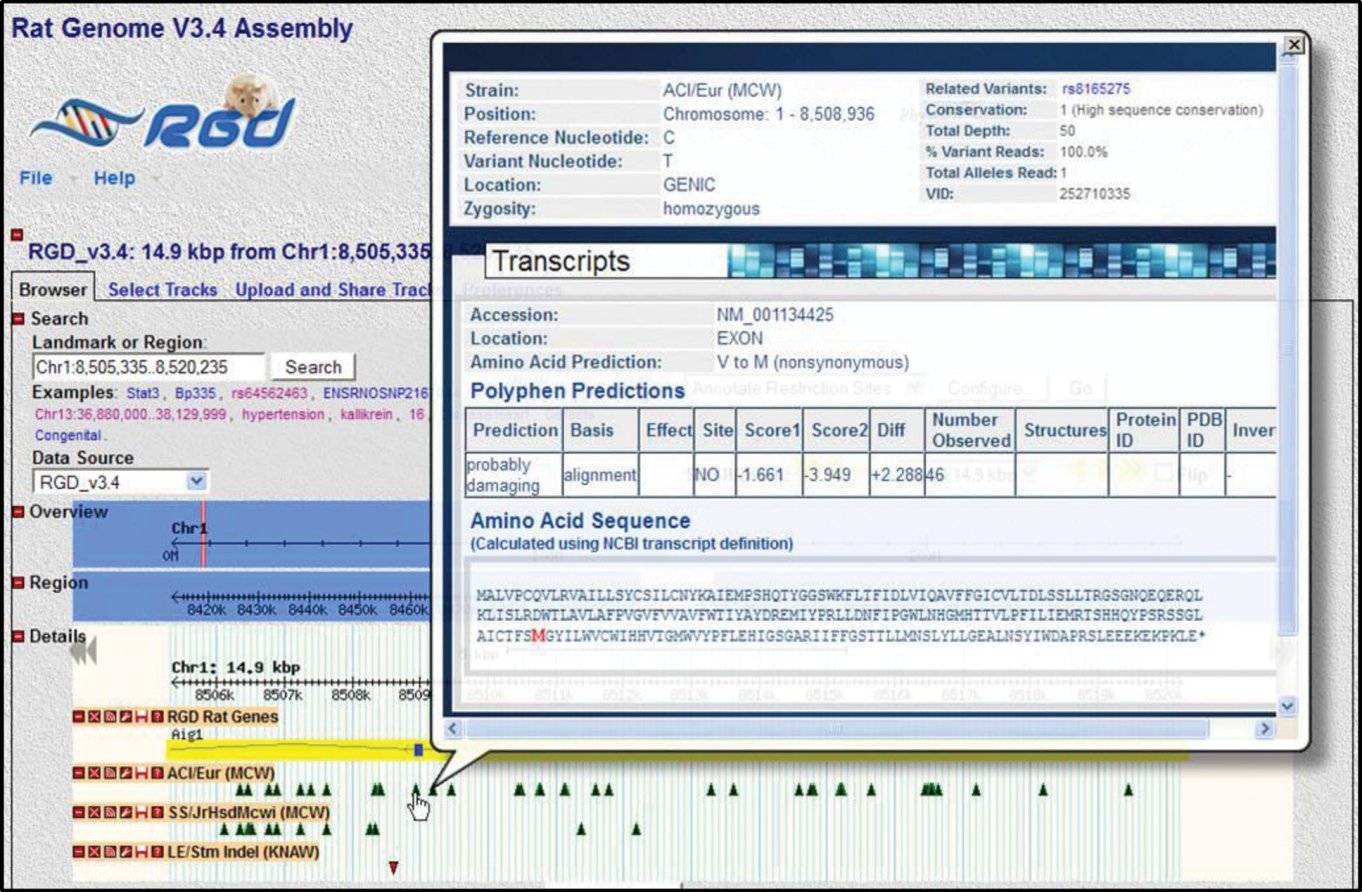
Variant details available on the Genome Browser. RGD's Rat Genome Browser (GBrowse) contains tracks for a variety of genomic elements, including genes, QTL, congenic strains, markers and strain-specific variants. Informative popups for the latter display information about the type, location and predicted consequences of each variant, as well as information pertaining to the sequence data supporting that variant call.

RGD has expanded its capacity to accommodate the ever-increasing amount of data generated by researchers using next-generation sequencing technologies and has incorporated copy number variations, single nucleotide polymorphisms (SNPs), indels and mutations into its reports and tools. To provide timely access to strain-specific variants generated from whole genome sequencing (WGS) projects, RGD developed a pipeline for data submitted by researchers for display on its Genome Browser and to date this has resulted in 40 single nucleotide variation (SNV) tracks, nine indel tracks, a copy number variation track and tracks for variation data from dbSNP (11) (http://www.ncbi.nlm.nih.gov/SNP/) and Ensembl (12) (http://www.ensembl.org/index.html). These include tracks from similar substrains, allowing users to compare results from different platforms and analysis systems. Variant density plots are available for larger regions as well as individual variation tracks with popups outlining details for a variant such as depth, conservation, location, zygosity and prediction of effect (Figure 1) for smaller regions. The human Genome Browser at RGD also provides tracks for 1000 Genomes Data (13) (http://www.1000genomes.org/) and automated pipelines incorporate variants identified as clinically important from ClinVar (http://www.ncbi.nlm.nih.gov/clinvar/). Rat gene report pages provide links to lists of variants reported in various strains as well as one-click access to those data in the Variant Visualizer (described below), while human gene reports detail clinically significant variants with links to individual variant reports (Supplementary Figure S1). Adjustments to QTL and strain reports and the creation of SNP report pages accommodate the increasing use of SNPs as position markers for identification of QTL and the generation of congenic strains.

### Variant Visualizer

**Figure 2.**
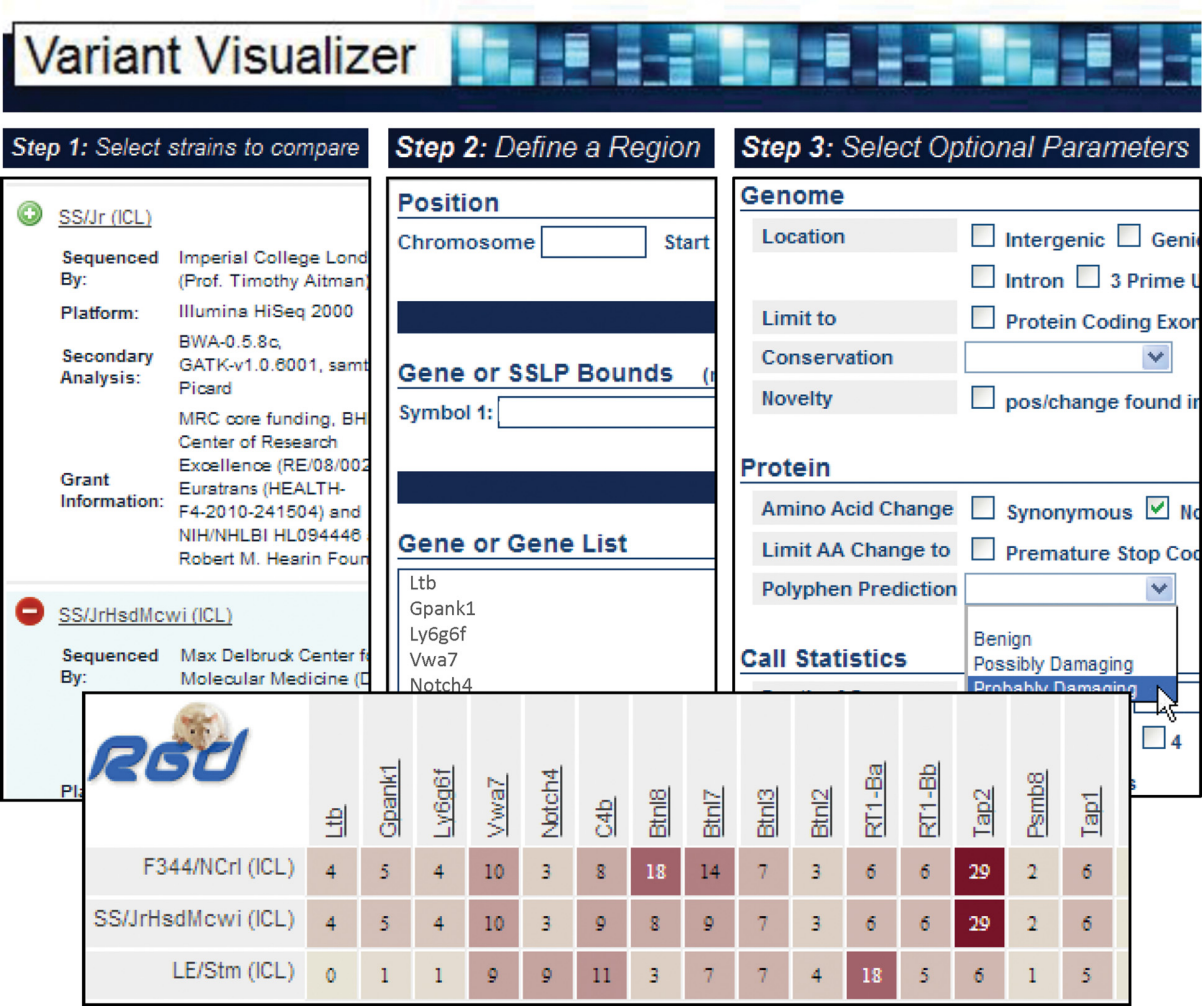
Search and filter options for variant types in Variant Visualizer. After selecting the genomic assembly and the strains of interest (upper left), and specifying the region, gene or list of genes of interest (upper middle; in this case, a list of genes was entered), the user has the option of filtering the results by the type of variant, its location relative to genes or transcripts, and the call statistics (upper right). If no selections are made on this page, the tool will return all of the variants that meet the input strain and region criteria. Once these selections have been made, the ‘Variant Distribution’ view (lower) shows the number of variants for each gene in which at least one of the strains queried contains at least one variant matching the criteria.

**Figure 3.**
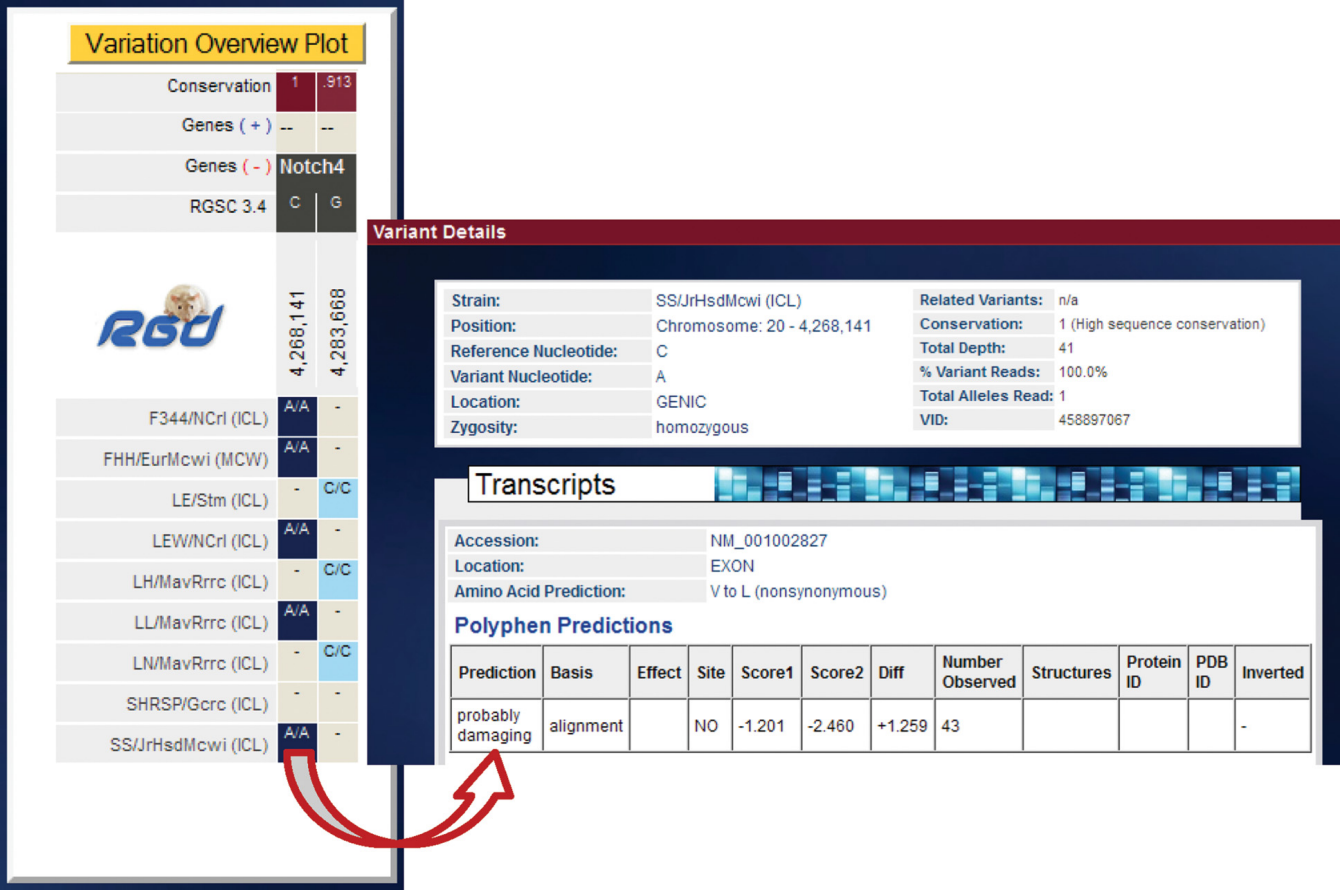
Variant Visualizer results showing location and type of variant within gene structure. Clicking on a gene symbol in the ‘Variant Distribution’ view opens the ‘Variation Overview Plot’ for that gene. Clicking a specific variant opens the variant detail display showing the type, location and predicted consequences of the selected variant, the calculated conservation of that nucleotide across species and information about the sequence data supporting the variant call, such as the read depth.

RGD created the Variant Visualizer to provide visual representation of variations across multiple strains for single and multiple genes or larger genomic regions. Strain-specific variants submitted to RGD are incorporated into the Variant Visualizer as well as the Genome Browser. The query tool (Figure 2) provides users with multiple options to search by combinations of strains and a single gene, a gene list or a genomic position. Users can then filter by variant location, type of amino acid change or call statistics. For sets of genes, results indicate the number of variants of the chosen type for each gene for the selected strains. A click on the gene takes the user to a full gene view showing the sequence and position within the gene for each variant. Clicking on a variant returns a detail view of each variant including PolyPhen predictions (Figure 3). For consistency, this detail view is the same as that described earlier for strain-specific variants in the rat Genome Browser (Figure 1).

## PHENOTYPES AND DISEASES

### PhenoMiner

**Figure 4.**
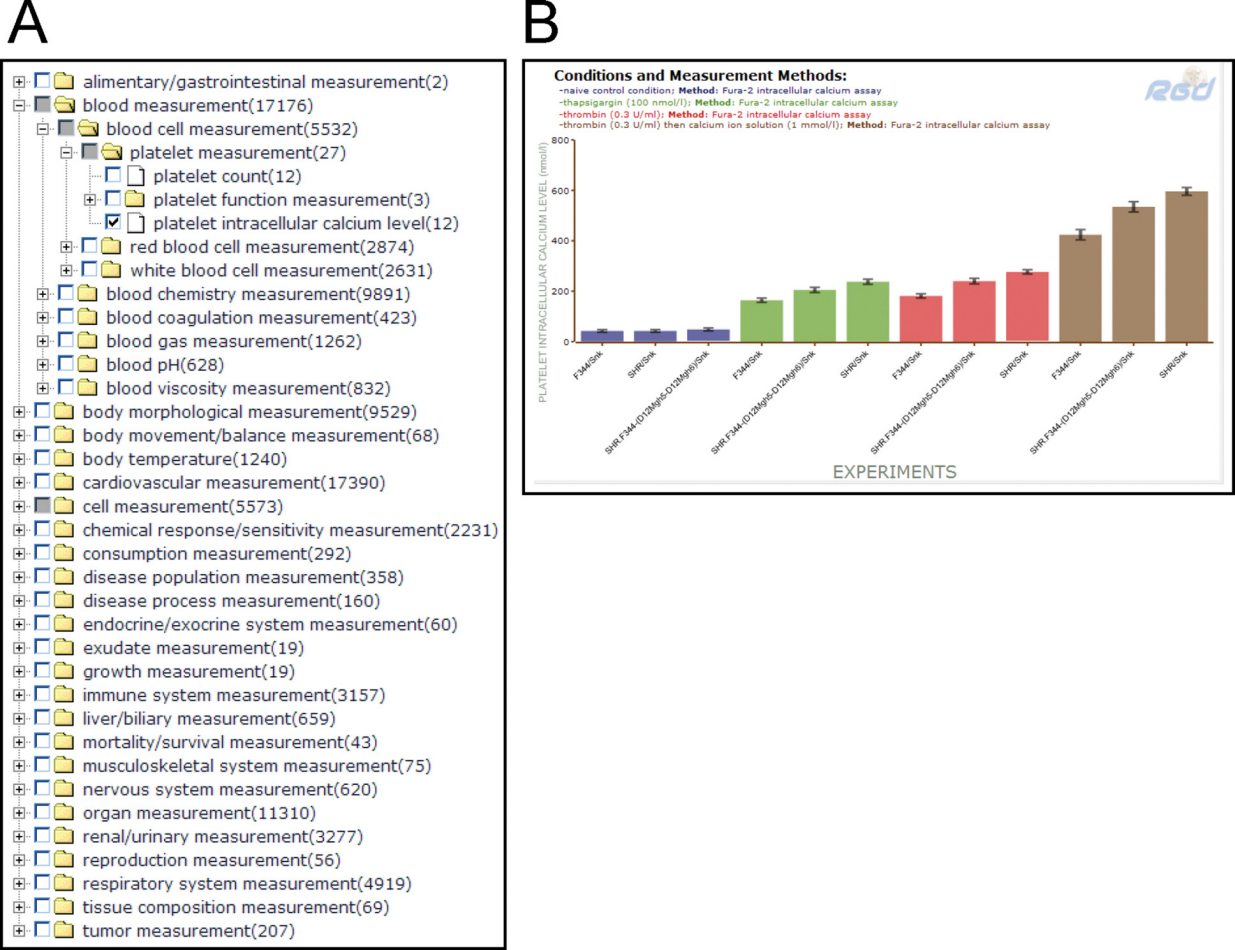
(**A**) Categories of phenotype measurement records in PhenoMiner. RGD's Clinical Measurement Ontology is a hierarchical vocabulary of specific measurements used both clinically and in the research laboratory. Users can choose a higher level term to see results for all the measurements in that category, or ‘drill down’ to find a specific measurement of interest, such as ‘platelet intracellular calcium level’. (**B**) PhenoMiner bar chart results for ‘platelet intracellular calcium level’ comparing values in untreated control rats versus rats treated with drugs such as thapsigargin and thrombin. The display makes it easy to compare across strains, conditions and methods, either within a single study or across multiple studies. In addition, the specific quantitative data can be downloaded for further analysis or comparison with a researcher's own results.

An important part of translational research involves connecting genetic variation to variations in phenotype. The availability of multiple strains exhibiting a wide range of phenotypes makes the rat an ideal model for such studies. While the incorporation of strain-specific variations into multiple tools at RGD has provided users with the means to create genomic profiles for individual strains, the PhenoMiner project is a companion initiative to integrate phenotype measurement data from multiple experiments to provide both a comprehensive view of phenotype variation across strains and profiles for individual strains (8). RGD uses multiple ontologies to standardize these data for integration, including the Rat Strain Ontology (14), Clinical Measurement Ontology, Measurement Method Ontology and Experimental Condition Ontology (7,15). These ontologies, along with other fields, provide the ability to specify (i) sample information: strain, sex, sample number, age, (ii) measurement information: what was measured, value, units, average type, standard error, standard deviation, (iii) method information: method type, site of measurement, duration of measurement, insult type and post insult time, (iv) conditions under which the measurement was taken: condition type, value and units, application type, ordinality to indicate simultaneous and sequential conditions. Data are incorporated from large-scale phenotyping projects such as the PhysGen Program for Genomic Applications (16) and the National BioResource Project (NBRP) for rat in Japan (17), curated from the literature and directly submitted by researchers. A recent curation initiative resulted in the integration of all phenotype measurement data in published rat QTL papers. PhenoMiner currently has over 50 000 records covering measurements across the physiological and morphological spectrums (Figure 4A). Users can build queries beginning with strains, clinical measurements, measurement methods or experimental conditions to provide filtering for the specific set of records desired. The user-friendly interface presents options based on previous choices so that for example, when strains are chosen, the user is only presented with the clinical measurement options for which records are available for those strains. Results from the query are presented in several forms. Bar charts are presented for each clinical measurement chosen (Figure 4B) and users can further customize this view by deleting bars or removing results based on strain, experimental conditions or measurement methods or by age or sex. Downloadable tables for all records returned also give the user options for sorting data by various parameters. Individual strain report pages provide a summary of clinical measurement records available for that strain with direct links to the bar charts and downloadable tables at PhenoMiner.

### Annotations and portals

RGD has continued its disease focus through targeted curation initiatives and Disease Portals (2,5). Disease areas are identified and prioritized by the number of related rat publications, funded grants and the interests of the rat research community and funding agencies. There are currently nine portals for renal diseases, cancer, cardiovascular diseases, diabetes, obesity and metabolic syndrome, respiratory diseases, neurological diseases, immune and inflammatory diseases, and diseases of the sensory organs. The portals include data related to the diseases of interest including genes, QTL and strains, pathways, and biological processes. Disease association annotations are created through manual curation as well as data imports from OMIM (18), the Genetic Association Database (19) and ClinVar (20). The Ontology Browser (6) also provides easy access to disease-related genes, QTL and strains with reports illustrating positions across the genome, links to the Genome Browser, or GBrowse, and data download options. Report pages such as those for genes have both quick lists of associated diseases and table views (Supplementary Figure S2), with human gene reports providing information on individual variants (see Supplementary Figure S1). Disease track options in the Genome Browser allow users to access and filter genes within a regional view by disease association.

## ENVIRONMENT AND EXPERIMENTAL CONDITIONS

As the rat is widely used in pharmacology and toxicology studies, RGD has focused on the acquisition of data of importance to these research communities. As part of the PhenoMiner project described above, nearly 10 000 phenotype measurement records were added in which drugs, chemicals or radiation were part of the experimental conditions. As a complement to such phenotype data, over 2.4 million chemical–gene and drug–gene interaction annotations were imported from the Comparative Toxicogenomics Database for rat, human and mouse genes (21) using the Chemical Entities of Biological Interest (ChEBI) ontology to standardize annotations (22). Gene–drug/chemical tracks on the Genome Browser provide additional access to these data and allow users to filter genes within a region by type of gene–drug/chemical interaction. Using the Ontology Browser or keyword search, a search retrieves a report detailing genes, QTL and phenotype measurement records associated with that drug or chemical.

**Figure 5.**
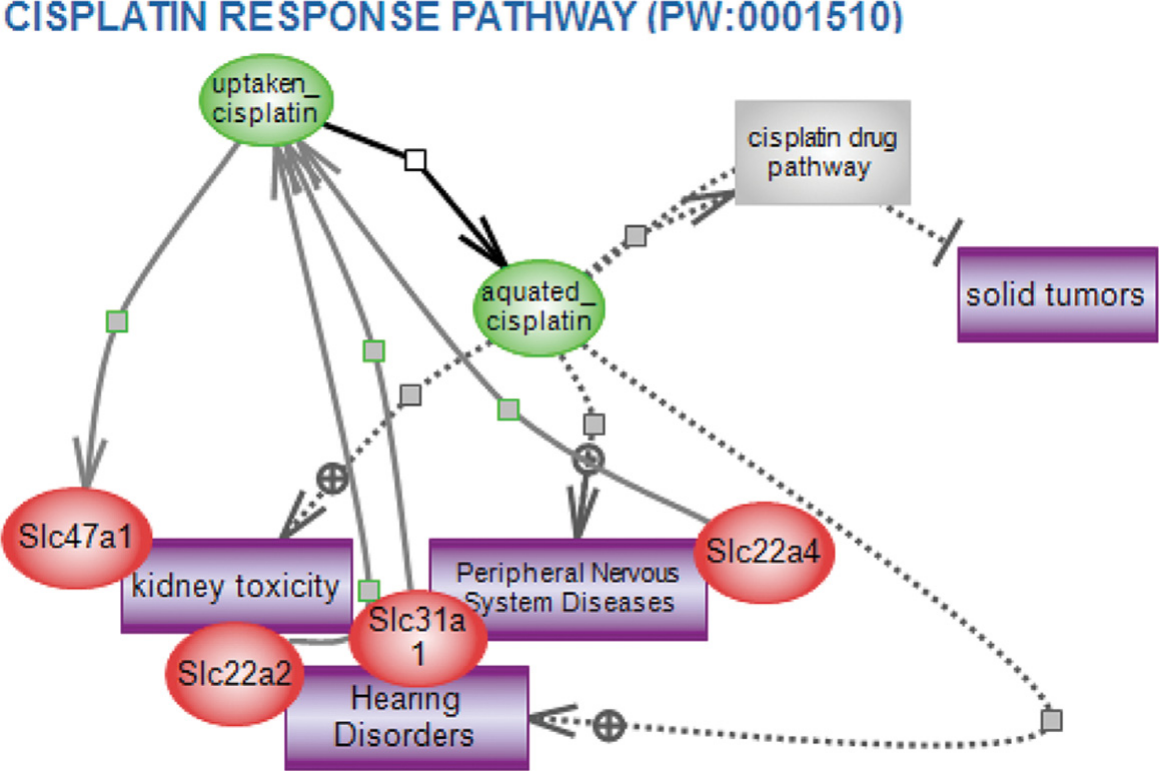
Cisplatin response pathway. RGD's interactive pathway diagrams give a detailed and intricate view of a growing list of regulatory, signaling, metabolic, disease and drug pathways. The page begins with an overview of what is known about that pathway (not shown in this figure), and a diagram showing the various players and their interactions and relationships in the functioning of the pathway. Disease pathways and altered pathways show the details of what can go wrong with a specific interaction to cause a breakdown in the function of the network. Pathway pages also give information about the other pathways, diseases and phenotypes with which the members of a particular pathway are associated.

The focus on environment and experimental conditions has also extended to the pathway portal at RGD (9,10) with expansions of regulatory and drug pathway diagram pages. These include those related to hypoxia, stress response, titanium dioxide nanoparticle response and toxic response to drugs such as acetaminophen and cisplatin (Figure 5). RGD also provides an expanding set of pharmacokinetic and pharmacodynamics pathways for drugs used to control diabetes, cardiovascular diseases and cancer. These and all pathway diagrams are manually built using the research literature for context and the Pathway Studio software from Elsevier for graphics; the diagram pages are created using a web application tool built at RGD (9,10).

## INTERACTIONS AMONG VARIATIONS IN GENOME, PHENOTYPE AND ENVIRONMENT

The increasing availability of genomic, phenotype and environmental variation data at RGD led us to develop several projects designed to illustrate and analyze the interactions among these data types.

### QTL

As part of the effort to integrate phenotype measurement data from QTL literature, all QTLs were re-annotated with ontologies to standardize strain, trait (23), clinical measurement, measurement method and experimental condition and provide direct links to experimental data generated as part of the QTL experiment. These annotations on the QTL reports provide direct links to the respective ontology report pages providing users with easy access to related data. A link to a strain ontology report page returns a list of QTL in which the strain was used as well as related strains and substrains and their QTL. The ontology reports linked to clinical measurement, measurement methods and experimental conditions also provide details on related QTL, strains and phenotype records providing the user with easy navigation between data types. The QTL search was modified to include ontology searches, allowing the user to search and filter by strain and related strains, vertebrate trait, measurement type and method and experimental condition. The QTL report provides details on genes, markers and other QTL within the region with download functions and links to individual reports. The gene list can be easily input into the Variant Visualizer to view variants of interest between the two strains used in the experiment.

### GViewer

RGD's Genome Viewer (GViewer) provides a genome-wide view of mapped genomic elements such as genes, QTL and congenic strains related to simple and complex ontology searches. Users can also layer on additional data returned from their initial searches using data types or ontology terms. With the re-annotation of QTL using ontologies for strain, trait and experimental data and the inclusion of gene–drug/chemical interactions in RGD, users can now create Boolean searches with terms from the following: Gene Ontology (24), MEDIC disease vocabulary (25), NeuroBehavioral Ontology (26), Mammalian Phenotype Ontology (27), Pathway Ontology, Clinical Measurement Ontology, Measurement Method Ontology, Experimental Condition Ontology, Vertebrate Trait Ontology and ChEBI. This provides another connection between genomic elements, phenotypes and experimental data. For example, users can easily see genes and QTL associated with particular diseases, phenotypes and specific drugs or chemicals.

### Gene Annotator

**Figure 6.**
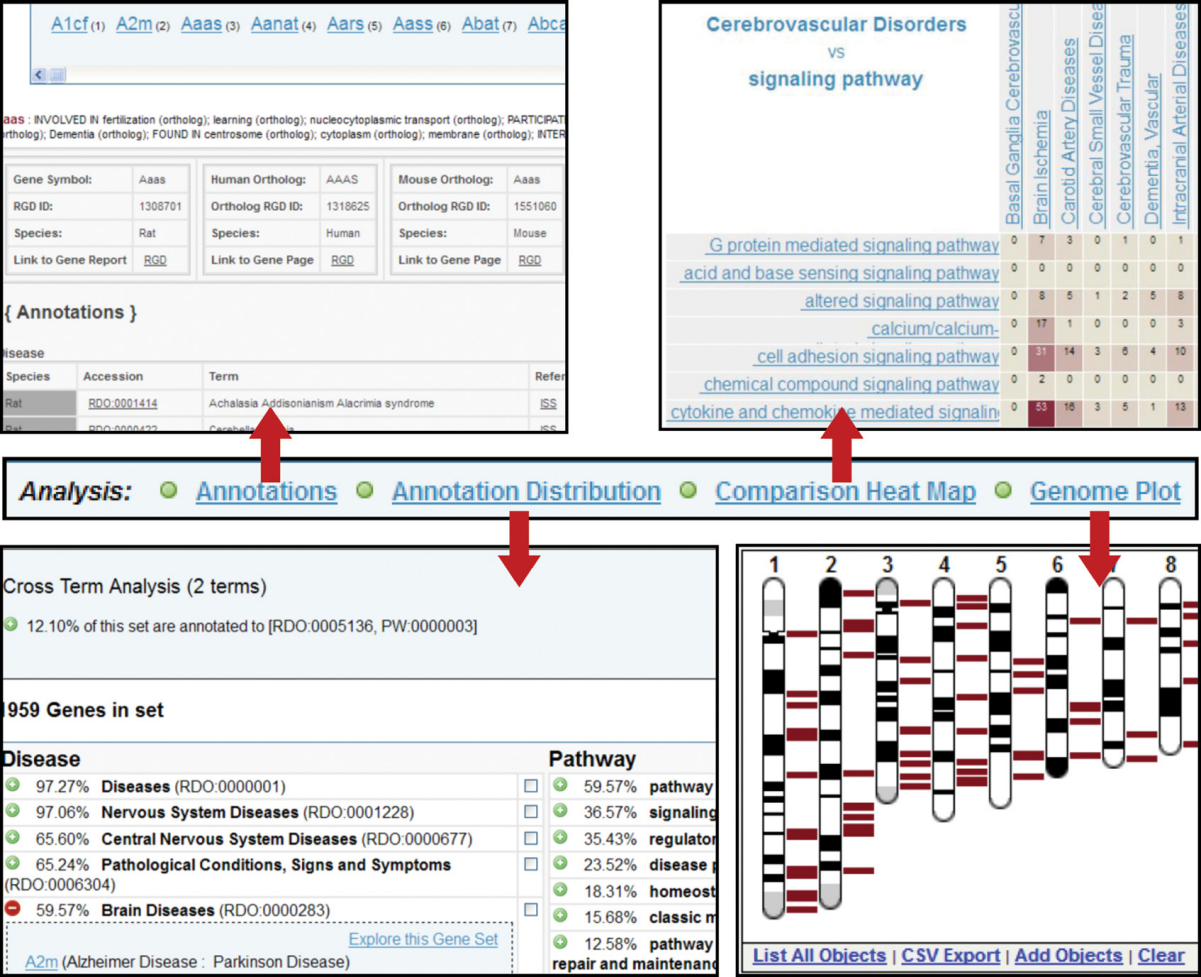
The Gene Annotator Tool. Shown in the center of the figure is the menu bar from the Gene Annotator (GA) tool. The default result is the ‘Annotations’ page (top left), which gives detailed lists of annotations for each gene in the input list and its corresponding orthologs, as well as a list of external database identifiers for that gene with links to additional information at the other databases. The ‘Annotation Distribution’ analysis (bottom left) indicates the percentage of genes in the input list associated with lists of disease, pathway, phenotype, biological process, molecular function, cellular component and chemical interaction terms, beginning with the terms that appear most commonly. Selecting a term shows the subset of the input list of genes that are associated with that term or any more specific term beneath it in the ontology. Check boxes allow the user to select multiple terms within one or across multiple ontologies to see the genes with annotations to all the selected terms. This smaller subset of the original list can then be entered into the GA Tool for further analysis. The ‘Comparison Heat Map’ function (top right) allows users to select any two ontologies, or to view the overlap between two branches of the same ontology. In this case, the number of genes from the original input list which are associated with disease categories under ‘Cerebrovascular Disorders’ and pathway categories under ‘signaling pathways’ are shown, with intersections containing a higher number of associated genes displayed as increasingly darker colors. Finally, the ‘Genome Plot’ (bottom right) shows the location of each gene in the list against the full set of chromosomes for the species, in this case, the rat karyotype, with the chromosomal positions for all the genes in the list presented in a table below the image (not shown). Functionality for the Genome Plot is the same as that described earlier for the Genome Viewer tool.

The Gene Annotator or GA Tool is a one-stop functional analysis tool for rat, human and mouse genes. For any of the three organisms, users can upload a list of common identifiers from RGD, EntrezGene, GenBank, Ensembl and Affymetrix or search by a chromosomal region or functional ontology identifiers to retrieve comprehensive reports for each gene. The output (Figure 6, top left) contains data related to disease and phenotype, pathway, Gene Ontology, and drug or chemical interactions, as well as dozens of identifiers and links to other sources. Functional analysis of the entire gene list or subsets can be accomplished through the Annotation Distribution Tool and the Comparison Heat Map. The Annotation Distribution Tool (Figure 6, bottom left) provides a dynamic assessment of the functional make-up of the gene list, showing the percentage of genes associated with various diseases, pathways, biological processes and functions. Users can retrieve the genes associated with a particular disease, pathway or function and further analyze this subset for functional commonalities. The tool also facilitates the retrieval of genes associated with multiple functional categories, such as those associated with a particular disease, a set of pathways and a class of drugs. The Comparison Heat Map (Figure 6, top right) visualizes the distribution of genes across two functional parameters such as disease and pathway. Users can choose the ontologies displayed on the X and Y axes and see the number of genes from their original list in the intersection of classes from each of the categories. Leveraging the power of the ontologies, users can expand each category with a click to return more specific categories and the genes with annotations in the cross-section of these. The Genome Plot (Figure 6, bottom right) provides a genome-wide view of positions for genes in the set, as well as the ability to overlay other data such as QTL, in order to see the overlap. For reference, chromosomal positions are listed in a table below the image. The plot also provides direct links to GBrowse where users can add other tracks such as SNPs, QTL, disease or transcripts. Links from the Variant Visualizer and GBrowse to the reports in the GA Tool provide direct access to comprehensive multiorganism functional profiles.

## SOFTWARE IMPLEMENTATION AND DATA ACCESS

All tools mentioned are built in-house by RGD developers except GBrowse (28). RGD tools are built on J2EE technologies (http://java.sun.com/j2ee/overview.html) and driven off the RGD Oracle database. The tools can be run on any Java container that implements the Java Servlet and JSP (JavaServer Pages) specification. The popular Spring (29) framework's MVC (model-view-controller) architecture streamlines the application web development. The user interface relies heavily on Ajax and Javascript along with CSS (Cascading Style Sheets). Supported browsers include Internet Explorer, Firefox, Chrome and Safari.

An important avenue for RGD data access is the FTP site (ftp://rgd.mcw.edu/pub/). All of the data found on the RGD website can be downloaded in bulk from the FTP site. Files available for download include descriptive information for genes, QTL and strains, functional gene annotations, SSLPs, SNPs, ontology term files and more. Bulk downloads of data allow researchers to perform analyses with their own software tools or other tools that are not available at RGD.

## DISCUSSION

RGD continues to acquire, curate and integrate data of critical importance to disease research communities. These include datasets associated with the interacting factors in disease processes—genomic variation, environment, molecular pathways and the resulting phenotypes that define disease. Increasing emphasis on the development of tools that integrate, analyze and visualize multiple types of data from rat, human and mouse studies has made RGD a comprehensive research platform for disease and cross-species investigations.

## SUPPLEMENTARY DATA

Supplementary Data are available at NAR Online.
